# Clinicopathological characteristics of light chain proximal tubulopathy in Korean patients and the diagnostic usefulness of immunohistochemical staining for immunoglobulin light chain

**DOI:** 10.1186/s12882-020-01813-w

**Published:** 2020-04-23

**Authors:** Minsun Jung, Youngeun Lee, Hajeong Lee, Kyung Chul Moon

**Affiliations:** 1 Department of Pathology, Seoul National University Hospital, Seoul, 03080 Republic of Korea; 2 Department of Internal Medicine, Seoul National University Hospital, Seoul, 03080 Republic of Korea; 3 Kidney Research Institute, Medical Research Center, Seoul National University College of Medicine, Seoul, 03080 Republic of Korea

**Keywords:** Immunoglobulin light chains, Immunohistochemistry, Light chain proximal Tubulopathy, Monoclonal Gammopathy, Multiple myeloma

## Abstract

**Background:**

Light chain proximal tubulopathy (LCPT) is a rare paraproteinemic renal disease that has been mostly reported in Western patients. LCPT is characterized by the accumulation of immunoglobulin (Ig)-light chain (LC) in the proximal tubule. Immunohistochemical staining for Ig-LC has not been investigated in the context of LCPT. We reported the clinicopathological characteristics and Ig-LC immunoexpression of patients with LCPT for the first time in Korea.

**Methods:**

We reviewed the clinicopathological findings of 5 Korean patients diagnosed with LCPT between 2016 and 2018. In addition, immunohistochemical staining for κ-LC and λ-LC was conducted on paraffin-embedded tissues.

**Results:**

The median age was 63 years, and the male-to-female ratio was 3:2. The primary renal manifestations were either azotemia or tubular proteinuria. All patients were diagnosed with multiple myeloma with monoclonal κ-LC (#1–2) or λ-LC (#3–5) in the serum and urine. Kidney biopsies revealed diverse and subtle alterations of the proximal tubule, including crystallization, vacuolization, and/or swelling. Electron microscopy revealed crystals in patients #1–2 and non-crystalline particles within numerous/large/dysmorphic lysosomes in patients #3–5. Ig-LC restriction was demonstrated in the proximal tubule as κ-type in patients #1–2 and as λ-type in patients #3–5 by immunohistochemistry and immunofluorescence. Immunohistochemical staining showed diffuse positivity to κ- and λ-LC, although immunofluorescent staining for κ-LC was focal and weak. LCPT has diverse clinicopathological characteristics and subtle morphological alterations, which necessitate ancillary tests for diagnosis.

**Conclusions:**

We introduced immunohistochemical staining for Ig-LC as a useful tool for the diagnosis of LCPT, especially in the case of κ-type crystals.

## Background

Monoclonal light chain (LC), when overproduced in multiple myeloma (MM) and other paraproteinemic hematologic disorders, can affect the renal tubule [1]. For example, monoclonal immunoglobulin (Ig)-LC precipitates with Tamm-Horsfall protein in the distal tubule of the kidney, resulting in cast nephropathy which is the most common renal disorder of MM [1, 2]. Recently, light chain proximal tubulopathy (LCPT) has been emphasized as a distinct entity of LC-related kidney diseases. LCPT has been typified by the crystallization of κ-LC in the proximal tubule, which is associated with Fanconi syndrome in approximately 40% of patients [2, 3]. LCPT without crystal, which was described more recently, is associated with either κ- or λ-LC accumulation [2, 4]. LCPT can be an early manifestation of an underlying hematologic disorder [2, 5–7], and prompt treatment can prevent permanent renal impairment [2, 8]; therefore, it is important to properly diagnose LCPT. However, the clinicopathological characteristics of LCPT are not fully defined, which makes the diagnosis challenging [7]. Moreover, although immunofluorescence (IF) and electron microscopy (EM) are frequently utilized in the diagnosis process, immunohistochemical (IHC) staining for Ig-LC has not been investigated in the context of LCPT. Here, we report the clinicopathological characteristics of 5 Korean patients with LCPT and describe the diagnostic implications of IHC staining for Ig-LC.

## Methods

We retrieved 5 kidney biopsy samples from patients diagnosed with LCPT at Seoul National University Hospital between 2016 and 2018. Any patients who showed evidence of paraprotein-induced glomerular disease, amyloidosis, or cast nephropathy were excluded [1, 4, 7]. All specimens were routinely evaluated using light microscopy (LM), IF, and EM. In addition, IHC staining for κ-LC (1:500; L1C1; Leica, Wetzlar, Germany) and λ-LC (1:1500; HP-6054; Leica) was conducted on paraffin-embedded tissues using the Benchmark autostainer (Ventana, Tucson, AZ) following the manufacturer’s instructions. The extent of histologic alterations in the proximal tubule, including acute tubular injury (ATI) and interstitial fibrosis and tubular atrophy (IFTA), was measured based on LM as absent, present but minimal (< 10%), or present (> 10%). The IF and IHC staining results were evaluated as either focal (< 50%) or diffuse (≥50%). Laboratory data and follow-up information were obtained from medical records. The median follow-up period was 20 months (range, 4–24 months). Biopsies were performed within 1 month after the detection of monoclonal gammopathy. The diagnosis and treatment response of MM were based on standard criteria [9, 10]. MM stage was evaluated according to the International Staging System (ISS) [11].

## Results

The clinical features of patients with LCPT are summarized in Table 1. The patients’ ages ranged from 51 to 74 years. Three patients were men and two were women. Patients #1 and #3–4 had preexisting hypertension, and patients #3–4 had diabetes mellitus. The primary renal manifestations were either azotemia in 3 patients or tubular proteinuria in 3 patients, both of which were present in patient #1 (Table 1). Features of Fanconi syndrome, including glycosuria, hypouricemia, hypophosphatemia, polyuria, acidosis, and hypokalemia, were not observed in any patients. In the hematologic evaluation, all patients were diagnosed with MM in ISS stage I (#3, 5) or II (#1, 4) (Table 1). Monoclonal Ig-LC was identified in the serum and urine as κ (#1–2) or λ (#3–5) with an increased or decreased κ-to-λ free LC ratio, respectively. All patients received combined chemotherapeutic regimens. Autologous stem cell transplantation was followed in patients #2 and 4. At the last check-up, complete (#2, 4) or partial (#1, 3, 5) hematologic remission was achieved, and all patients exhibited normalization/improvement of renal function. One patient (#3) died of unknown causes.

**Table 1:** Clinical features and treatment outcomes of patients with light chain proximal tubulopathy

	Case #1	Case #2	Case #3	Case #4	Case #5
Renal presentation	Azotemia and proteinuria	Azotemia	Azotemia	Proteinuria	Proteinuria
Renal baseline					
SCr (mg/dl)	2.10	1.93	1.88	1.06	1.00
eGFR^a^ (ml/min/1.73 m^2^)	31.0	27.3	35.5	52.5	75.3
UPCr	3.64	1.64	0.89	4.26	1.39
Hematologic Dx	MM	MM	MM	MM	MM
BM plasma cell (%)	13.2	66.5	47.3	11.4	11.0
ISS	II	n.a.	I	II	I
Serum β2-microglobulin (μg/ml)	4.70	n.a.	3.01	4.26	2.79
Urine β2-microglobulin (μg/ml)	5.48	n.a.	0.61	0.46	n.a.
Monoclonal Ig	κ	G-κ	λ	G-λ	A-λ
Serum κ/λ ratio^b^	193.43	37.61	0.01	< 0.01	0.01
Urine κ/λ ratio^c^	1245.65	284.08	0.11	< 0.01	n.a.
FS^d^	Absent	Absent	Absent	Absent	Absent
Serum uric acid (mg/dl)	5.4	7.9	8.4	3.6	8.6
Serum phosphorus (mg/dl)	3.2	3.6	4.2	3.7	3.8
Treatment	VMP#9	VTD#5 ➔ SCT	VMP#2	VTD#3 ➔ SCT	VTD#5
Last f/u (month)	20	24	4	20	6
Hematologic outcome	PR	CR	PR	CR	PR
Renal outcome					
SCr (mg/dl)	1.26 (WNL)	0.72 (WNL)	1.44 (improved)	0.97 (WNL)	0.87 (WNL)
eGFR^a^ (ml/min/1.73 m^2^)	55.7 (WNL)	84.7 (WNL)	48.2 (improved)	58.0 (WNL)	88.3 (WNL)
UPCr	0.23 (improved)	< 0.2 (WNL)	< 0.2 (WNL)	< 0.2 (WNL)	0.25 (improved)

^a^Based on Modification of Diet in Renal Disease Study equation

^b^Normal range: 0.26–1.65

^c^Normal range: 2.04–10.37

^d^Glycosuria, hypouricemia, hypophosphatemia, polyuria, acidosis, and hypokalemia

*M* male, *F* female, *SCr* serum creatinine, *eGFR* estimated glomerular filtration rate, *UPCr* urine protein/creatinine ratio, *Dx* diagnosis, *MM* multiple myeloma, *BM* bone marrow, *ISS* International Staging System for Multiple Myeloma, *n.a.* not available, *Ig* immunoglobulin, *FS* Fanconi syndrome, *VMP* Velcade, Melphalan, prednisone, *VTD* Velcade, thalidomide, dexamethasone, *SCT* autologous stem-cell transplantation, *f/u* follow-up, *PR* partial remission, *CR* complete remission, *WNL* within normal limits

**Fig. 1:**
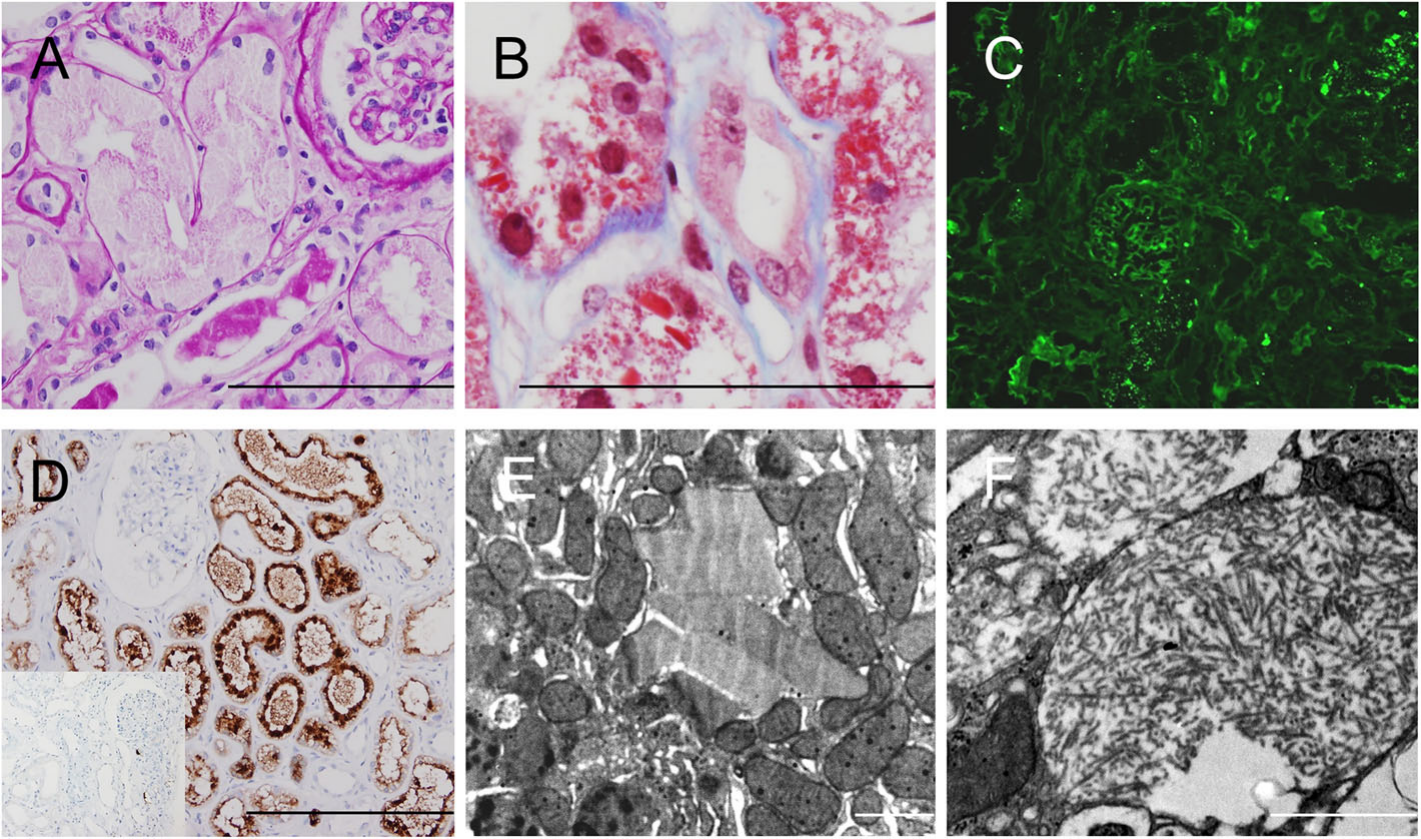
Microscopic findings of LCPT with crystal. **a** LCPT with crystal shows swollen tubules with abundant glassy cytoplasm along with an injured tubule that contains proteinaceous material in the lumen in patient #2 (PAS). **b** In addition, fuchsinophilic crystals are observed in the vacuolar cytoplasm of the tubules in patient #1 (MT). κ-LC is restricted in the tubular cytoplasm which is (**c**) weak and focal by IF but (**d**) diffuse by IHC (λ-LC in an inlet) staining in patient #1. EM analysis demonstrates (**e**, #1) rhomboid or (**f**, #2) needle-like crystals in the tubular cytoplasm with (**e**) occasional electron-dense deposits in the lysosome (left bottom). Scale bars indicate 10 μm in LM and 1 μm in EM figures

**Fig. 2:**
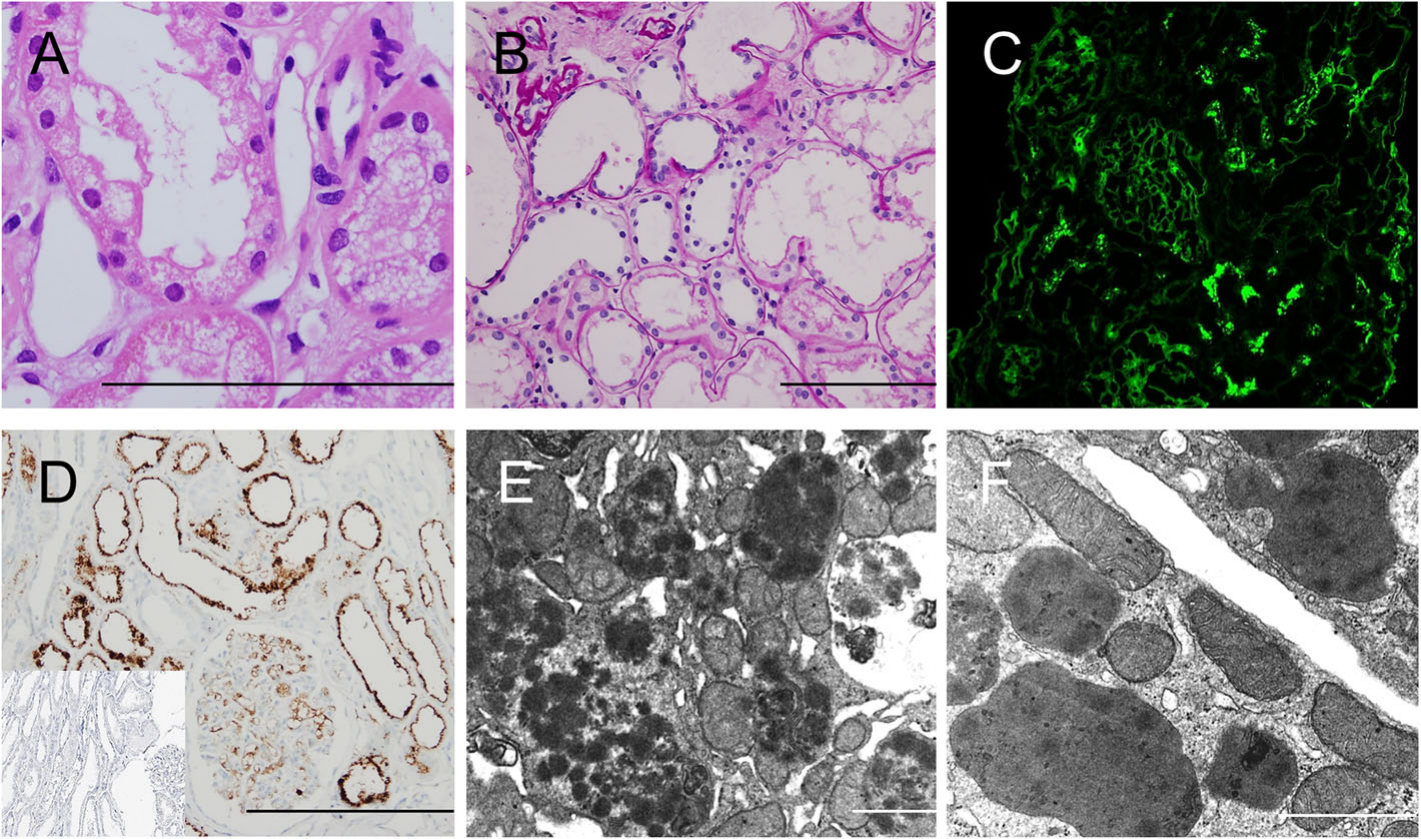
Microscopic findings of LCPT without crystal. LCPT without crystal shows (**a**) vacuolization (#3, HE) and (**b**) diffuse damage (#4; PAS) of the tubule. Diffuse accumulation of λ-LC is observed both in (**c**) IF and (**d**) IHC (κ-LC in an inlet) staining in patient #5. **e** The lysosome in the tubular cytoplasm is increased in number and contains numerous electron-dense deposits, which leads to mottled appearance in patient #4. **f** Large dysmorphic lysosomes with electron-dense particles are seen in patient #5. Scale bars indicate 10 μm in LM and 1 μm in EM figures

The pathological characteristics of the kidney biopsies are summarized in Table 2, Fig. 1, and Fig. 2. The proximal tubule was predominantly affected in all patients, exhibiting vacuolated and/or swollen cytoplasm with a glassy texture that was diffuse (#1–4) or focal (#5), as observed with LM (Fig. 1a, Fig. 2a). In addition, fuchsinophilic crystals were focally observed in patient #1 (Fig. 1b). Identical types of monoclonal Ig-LC in the system were detected by IF and IHC staining as follows: κ-type in patients #1–2, and λ-type in patients #3–5. The immunoreaction was focal for κ-LC (Fig. 1c) but diffuse for λ-LC (Fig. 2c) by IF; in contrast, diffuse Ig-LC restriction was detected by IHC in all patients (Fig. 1d, Fig. 2d). When observed with EM, the accumulated Ig-LC appeared as rhomboid-like (#1; Fig. 1e) or needle-like (#2; Fig. 1f) crystals in LCPT with crystal or as electron-dense particles in the lysosome in LCPT without crystal, which led to a mottled appearance (#3–5; Fig. 2e, f). Although similar deposits were occasionally observed in patients #1–2 (Fig. 1e), the lysosomes of patients #3–5 were numerous, large, and frequently dysmorphic (Fig. 2e, f). In addition to these changes, ATI (Fig. 2b) and IFTA were present in 5 and 4 patients, respectively. Luminal proteinaceous material (Fig. 1a) was present in varying degrees in 4 patients, and this material was reactive to both κ- and λ-LCs with similar intensity on IHC staining. The glomeruli and blood vessels were unremarkable, except for occasional global sclerosis and mild fibro-intimal thickening, respectively.

**Table 2:** Pathological characteristics of patients with light chain proximal tubulopathy

	Case #1	Case #2	Case #3	Case #4	Case #5
PTEC					
LM	Focal fuchsinophilic crystal, diffuse vacuolization and swelling	Focal vacuolization and diffuse swelling	Diffuse vacuolization and swelling	Diffuse vacuolization	Focal swelling
Ig-LC	κ	κ	λ	λ	λ
IF^a^	Focal	Focal	Diffuse	Diffuse	Diffuse
IHC^a^	Diffuse	Diffuse	Diffuse	Diffuse	Diffuse
EM	Crystal (rhomboid), lysosome with EDD	Crystal (needle-like), lysosome with EDD and focal dysmorphism	Increased/large/dysmorphic lysosome with mottled appearance	Increased/large/dysmorphic lysosome with mottled appearance	Increased/large/dysmorphic lysosome with mottled appearance
ATI^b^	Present, minimal	Present, minimal	Present, minimal	Present	Present, minimal
IFTA^b^	Present, minimal	Present, minimal	Present	Present	Absent
Tubular proteinaceous material	Present	Present	Absent	Present	Present
Glomerulus	GS (12.1%)	WNL	GS (12.5%)	GS (18.6%)	WNL
Blood vessel	Fibro-intimal thickening	WNL	Fibro-intimal thickening	Fibro-intimal thickening	Fibro-intimal thickening

^a^Focal IF or IHC staining: positivity in < 50% of the proximal tubule

^b^Minimal ATI or IFTA: 0–10% of the renal cortex

*PTEC* Proximal tubule epithelial cell, *LM* Light microscopy, *Ig-LC* Immunoglobulin light chain, *IF* Immunofluorescence, *IHC* Immunohistochemistry, *EM* Electron microscopy, *EDD* Electron-dense deposit, *ATI* Acute tubular injury, *IFTA* Interstitial fibrosis with tubular atrophy, *GS* Global sclerosis, *WNL* Within normal limit

## Discussion

We delineated the clinicopathological characteristics of patients with LCPT for the first time in Korea. LCPT accounted for 0.1–1.5% of native renal biopsies according to retrospective studies [4, 7]. Approximately 200 cases of LCPT have been reported, most of which have been from Western countries, with a small number of patients from Asian countries; LCPT has not been described in Korean patients [2–4, 7, 12–16]. Various plasma cell dyscrasias, such as MM in this report, can lead to the overproduction of free LCs, which are filtered by the glomeruli and reabsorbed in the proximal tubule [1, 17]. An accumulation of Ig-LC in the proximal tubule that exceeds the capacity of lysosomal proteolysis results in LCPT, and the chemodynamics of this Ig-LC are known to determine the clinicopathological features of LCPT [1, 7]. Thus, the detection of Ig-LC restriction in the proximal tubule is critical for the diagnosis of LCPT [7]. We demonstrated that IHC staining for Ig-LC was strongly positive for the identical Ig-LC detected by IF and even for κ-LC, which exhibited a limited reaction by IF. By forming a crystalline structure, κ-LC may evade immunoreaction in IF staining [7]. Although pronase treatment of the paraffin block has been suggested to enhance the immunoreactivity of IF staining for κ-type LC, this treatment is not always successful [7, 18]. Furthermore, immunogold analysis, which would be confirmative, is not widely available [7, 12]. The antigen retrieval step during IHC staining may enhance the immunoreactivity of Ig-LC. Therefore, we propose IHC staining for Ig-LC as a useful diagnostic tool for LCPT that would be complementary to or even a substitution for IF in patients with weak or inconclusive IF results.

This and previous reports on LCPT described tubule-centered pathological findings [2, 4, 7]. We detected fuchsinophilic crystals that were focally present in one of the two patients with LCPT with crystal; such crystals were previously reported to be observed with varying shapes and in varying degrees by LM in crystalline LCPT [2]. The presence of rhomboid-like or needle-like crystals under EM can confirm the diagnosis. Prior studies described the following variable histological features of LCPT without crystal: normal [4], cytoplasmic vacuolization [2, 7] or swelling [4, 7], tubular injury [2, 4, 7], or even necrosis [7]. In all patients in the present study, swelling and/or vacuolization of the proximal tubule were observed at least focally and were more apparent in crystalline LCPT than in non-crystalline LCPT [3]. In both types of LCPT, however, these morphological findings were generally subtle and easy to miss, which we ascribed to the promptness of systemic and renal work-ups. Therefore, a high degree of suspicion during the microscopic analyses and meticulous ancillary tests, including IF/IHC and EM, are required for the diagnosis [5]. The detection of numerous and sometimes dysmorphic lysosomes with a mottled appearance via EM was essential to diagnose LCPT without crystal in this and previous studies [2, 4, 7]. However, similar ultrastructural findings are shared by patients with LCPT with crystal to a lesser degree, which suggests that this feature is not entirely specific to LCPT without crystal. Moreover, these EM findings of the lysosome have been inconsistently included in the diagnostic criteria of LCPT without crystal in previous studies, which may explain the inconsistently reported proportion of non-crystalline LCPT, ranging from 13 to 77% [2, 4, 7, 8]. Because of the rarity and diverse pathological features of LCPT without crystal, the standardization of its diagnosis may warrant further investigations.

Consistent with prior studies [2, 4, 8, 12], renal insufficiency and/or tubular proteinuria were the main signs of LCPT, and they appeared to be similar between patients with crystalline and non-crystalline forms of LCPT. Signs of Fanconi syndrome were not observed in any patients. Although Stokes et al. suggested that patients with LCPT with crystal may have worse renal function and proteinuria and closer association with Fanconi syndrome than those with LCPT without crystal [2], we did not find such trends, which can be ascribed to the small number of patients in our study. The extent of ATI and IFTA was unlikely to be associated with initial and post-treatment renal function in this and previous reports [2]. Prompt and accurate diagnosis of LCPT was suggested to be important to prevent irreversible kidney dysfunction in patients with monoclonal gammopathy [1, 17]. MM therapy considerably improved the kidney function of patients with LCPT in the present report and previous reports [2, 8]. Luminal proteinaceous material, which was presumably an organized Ig-LC released from desquamated tubular epithelial cells [2, 3], can be a diagnostic pitfall. This material was differentiated from that of cast nephropathy by its location and the lack of multiple fracture lines or giant cell reaction [1]. Furthermore, it is reasonable to speculate that pathogenic LCPT without crystal needs to be differentiated from the physiological trafficking of overflowing LC with a benign nature [2, 4, 17].

## Conclusions

We reported the clinicopathological characteristics of 5 Korean patients with LCPT with or without crystal. The tubule-oriented microscopic features of LCPT are diverse and sometimes subtle. We introduced IHC staining for Ig-LC as a useful tool in the diagnosis of LCPT, especially in the case of κ-type crystals.

## Data Availability

All data generated or analysed during this study are included in this published article.

## Abbreviations

LC: Light chain
MM: Multiple myeloma
Ig: Immunoglobulin
LCPT: Light chain proximal tubulopathy
IF: Immunofluorescence
EM: Electron microscopy
IHC: Immunohistochemical
LM: Light microscopy
ATI: Acute tubular injury
IFTA: Interstitial fibrosis and tubular atrophy
ISS: International Staging System
